# Case Report: Eosinophilic gastritis with pyloric stenosis in immune dysregulation, polyendocrinopathy, enteropathy, X-linked syndrome

**DOI:** 10.3389/fped.2022.1039341

**Published:** 2022-11-21

**Authors:** Ronghua Yu, Yongmei Xiao, Wuhen Xu, Ting Zhang, Yizhong Wang, Hui Hu

**Affiliations:** ^1^ Department of Gastroenterology, Hepatology, and Nutrition, Shanghai Children’s Hospital, School of Medicine, Shanghai Jiao Tong University, Shanghai, China; ^2^ Molecular Diagnostic Laboratory, Shanghai Children’s Hospital, School of Medicine, Shanghai Jiao Tong University, Shanghai, China; ^3^ Gut Microbiota and Metabolic Research Center, Institute of Pediatric Infection, Immunity and Critical Care Medicine, School of Medicine, Shanghai Jiao Tong University, Shanghai, China

**Keywords:** IPEX, FOXP3, eosinophilic gastritis, pyloric stenosis, HSCT

## Abstract

Immune dysregulation, polyendocrinopathy, enteropathy, X-linked (IPEX) syndrome is a rare X-linked recessive immunodeficiency caused by mutations in the forkhead box protein 3 (*FOXP3*) gene. IPEX is characterized by the onset of intractable diarrhea, type 1 diabetes mellitus (T1DM), and eczema in the early stages of life. The typical clinic triad for IPEX is not always seen. Here, we report a 15-year-old male patient with atypical IPEX syndrome complicated with severe eosinophilic gastritis (EG) and pyloric stenosis. The patient had noticeable eczema during the first year of life and had a history of food allergies. At the age of 3 years, the patient was diagnosed with EG, *Helicobacter pylori* (HP) infection, pyloric stenosis with recurrent vomiting, and failure to thrive. The patient did not respond to long-term symptomatic treatments in the following years, including methylprednisolone, proton pump inhibitors (PPI), L-glutamine and sodium gualenate granules, anti-HP therapy, and balloon dilation. At the age of 12 years, the patient received surgical interventions, including a laparoscopic jejunostomy feeding tube placement, gastrojejunal anastomosis bypass, and jejunal-jejunal end-to-side anastomosis. Intractable diarrhea and T1DM were not present in the patient. At the age of 14 years, the patient was diagnosed with IPEX syndrome due to a c.748–750del (p.Lys250del) mutation in the leucine zipper domain of the FOXP3 protein. The patient underwent matched sibling peripheral blood hematopoietic stem cell transplantation (HSCT) and showed good evolution after 3 months of HSCT. In summary, this case report provides information of unusual gastrointestinal findings in IPEX syndrome and highlights the need for increased awareness and early diagnosis of IPEX syndrome, which is vital for improving the patient's outcome.

## Introduction

Immune dysregulation, polyendocrinopathy, enteropathy, X-linked (IPEX) syndrome is a rare X-linked recessive immunodeficiency characterized by the onset of intractable diarrhea, type 1 diabetes mellitus (T1DM), and eczema in the early stages of life. IPEX syndrome was first reported by Powell et al. in 1982 (1), and subsequent studies identified mutations in the forkhead box protein 3 (*FOXP3*) gene located in the centromeric region of the X chromosome as the genetic etiology (2, 3). Loss-of-function mutations in *FOXP3* lead to a defect of CD4^+^ CD25^+^ regulatory T (Treg) cell development, which impairs immune homeostasis and acts against autoimmunity (4, 5). IPEX syndrome is a fatal condition in which untreated affected individuals with typical clinical manifestations usually die within the first 2 years of life. IPEX syndrome may encompass other variables and distinct clinical manifestations that complicate the clinical diagnosis (6). The typical clinical triad for IPEX is not always seen. Many atypical cases, including late-onset symptoms, mild condition phenotypes, and other predominant clinical features, were diagnosed with the advent of next-generation sequencing (NGS) in recent decades, which indicates the variable expressivity of IPEX (6). Therefore, an early diagnosis is vital for patients with atypical IPEX syndrome receiving the appropriate treatments to achieve optimal outcomes.

Eosinophilic gastritis (EG) is a rare disorder characterized by marked eosinophilia and tissue damage in the stomach (7). The known etiologies of EG include infections of specific pathogens [such as *Helicobacter pylori* (HP)], drugs, connective tissue disorders, hematopoietic disorders, and food allergies (8). Eosinophilic infiltration occurs predominantly at different gastric wall layers, leading to diverse clinical manifestations (9). EG involving the muscle layer typically presents with symptoms of gastric outlet obstruction due to an increased thickness of the wall caused by eosinophil infiltration (10, 11). Here, we report a case with atypical IPEX syndrome complicated with severe EG and pyloric stenosis diagnosed by genetic testing at the age of 14 years.

## Case presentation

**Figure 1:**
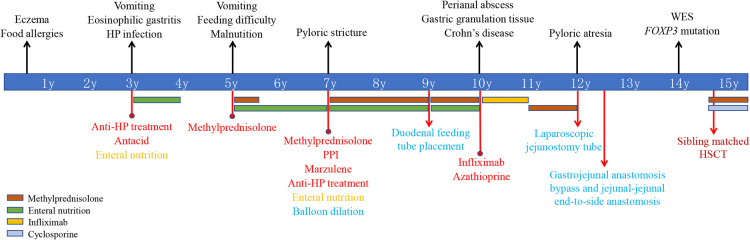
Timeline of clinical events, diagnostics, and treatments of the patient. *FOXP3*, forkhead box protein 3; WES, whole-exome sequencing; HP, *Helicobacter pylori*; PPI, proton pump inhibitors; HSCT, hematopoietic stem cell transplantation.

The patient was a 15-year-old boy with a genetic diagnosis of IPEX syndrome at the age of 14 years (Figure 1). The patient was born full-term by cesarean section with a birth weight of 4,200 g after an uneventful pregnancy. The patient is the fifth child of a healthy non-consanguineous couple of Chinese Han ethnicity. The patient's three old sisters are healthy. However, the patient's brother experienced intractable diarrhea, recurrent fever, and severe malnutrition, and died at the age of 2 years. The patient had noticeable eczema during his first year of life and had a history of food allergies in childhood. At the age of 3 years, he presented with recurrent vomiting and was treated with conventional antibiotics and symptomatic therapies in a local hospital for suspected acute gastroenteritis, but the symptoms did not improve. An esophagogastroduodenoscopy (EGD) showed multiple gastric ulcers, and a gastric biopsy revealed a marked infiltration of eosinophils and HP positivity. The colonoscopy was unremarkable. The patient was diagnosed with EG and HP infection and successively received anti-HP treatment, antacid medication, and nutritious formula feeding, but the patient's vomiting did not improve in the following 2 years. At the age of 5 years, the patient experienced frequent vomiting, an EGD showed multiple gastric ulcers, and a biopsy revealed eosinophil infiltration. No presence of duodenal villous blunting was observed. Although vomiting and gastric ulcers improved after 5 months of oral methylprednisolone, the patient started to present with feeding difficulties and malnutrition. The patient could not eat meat or rice because it caused vomiting. The symptom did not improve by symptomatic therapies and nutritional support in the following years.

**Figure 2:**
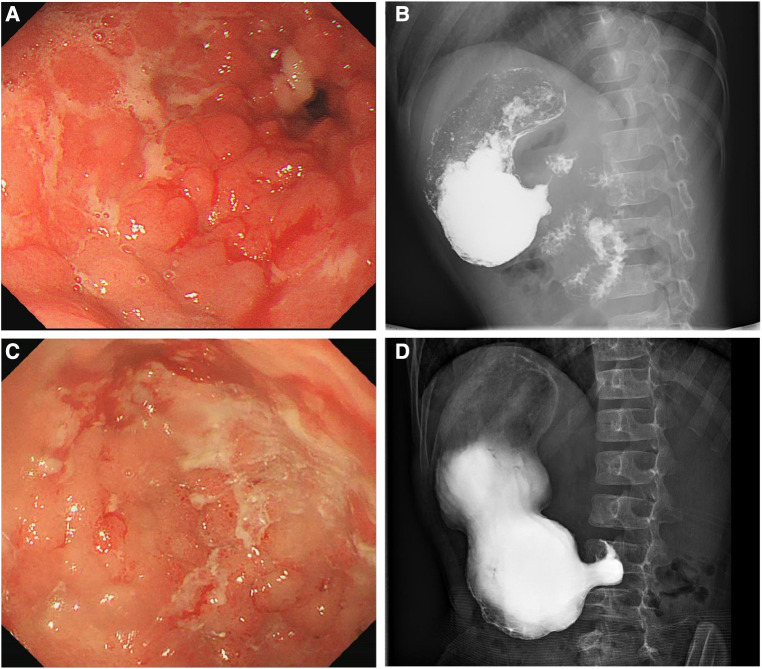
Esophagogastroduodenoscopy (EGD) showing edema, erosion, multiple ulcers, and pyloric stenosis of the stomach at 7 (**A**) and 12 (**C**) years old. Upper gastrointestinal radiography showing gastric retention and pyloric stenosis at 7 (**B**) and 12 (**D**) years old.

**Figure 3:**
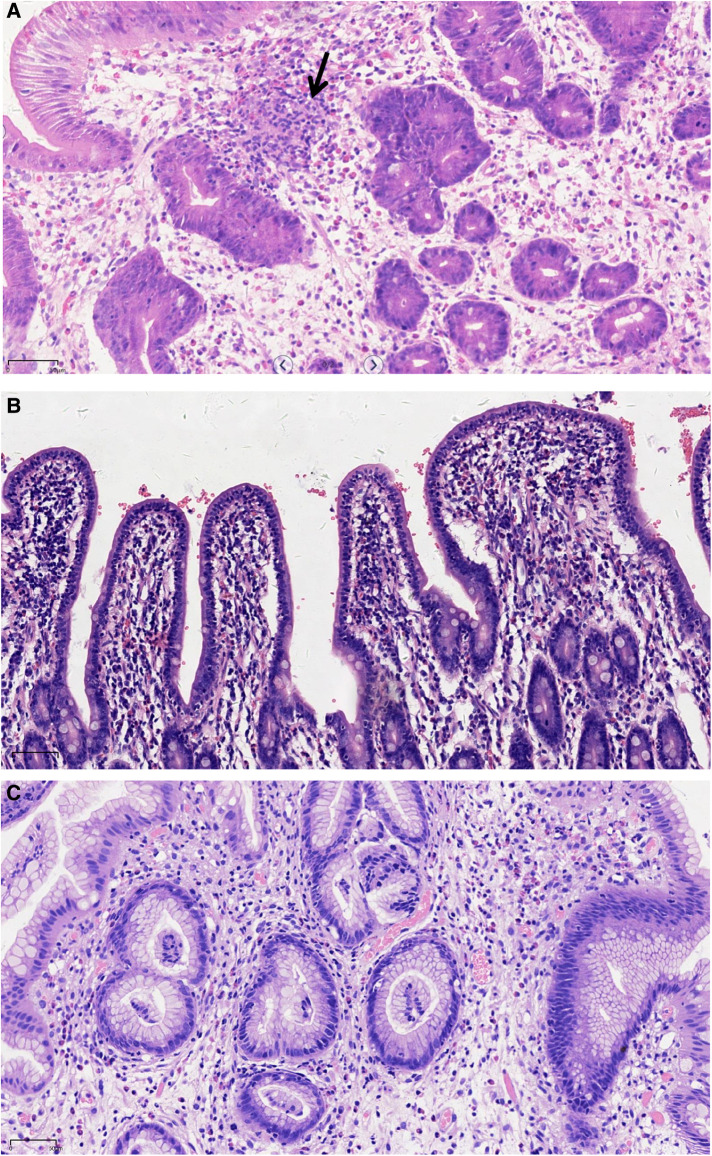
Representative histological images showing the infiltration of eosinophils, plasmocytes, and neutrophils in the upper gastrointestinal tract. (**A**) Gastric nonnecrotizing granulomas (arrow). (**B**) Small intestinal villi. (**C**) Gastric antrum (H&E, ×200; scale: 50 µm).

At the age of 7 years, the patient was admitted to our hospital for further evaluation. Physical examinations revealed severe failure to thrive (height, 107 cm, Z-score, −3.12; weight, 15.2 kg, Z-score, −4.22; BMI, 13.13 kg/m^2^, Z-score, −2.42) (Supplementary Figure S1). An EGD showed redness, erosion, and edema throughout the stomach. Pyloric stricture and stiffness were observed, and the endoscope could not enter the duodenal bulb (Figure 2A). Upper gastrointestinal radiography revealed pyloric stenosis with a slow barium passage (Figure 2B). A biopsy of the antrum suggested moderate non-atrophic gastritis, infiltration of eosinophils (10–30 eosinophils/HPF), and HP infection. The colonoscopy was normal, and no presence of villous blunting was observed in the terminal ileum. The laboratory blood tests were unremarkable, including normal eosinophil counts, complement, immunoglobulin G (IgG), IgA, IgM, and IgE levels. The serum tests of antineutrophil cytoplasmic and other autoantibodies, including antinuclear, antidiabetes-related (anti-insulin and anti-islet cell antibodies), and antithyroglobulin autoantibodies, were all negative. Balloon dilation was applied to treat the pyloric stenosis. The patient received oral methylprednisolone (4 mg per day), proton pump inhibitors (PPI), L-glutamine and sodium gualenate granules, and anti-HP treatment. However, the vomiting reoccurred after improvement, and the pyloric stenosis did not improve. In the following years, the patient was repeatedly admitted to our department. The patient was managed with a series of symptomatic and supportive treatments, including multiple balloon dilations, enteral nutrition, and oral methylprednisolone. At the age of 9 years, an EGD showed that the pyloric stenosis was aggravated. Endoscopically assisted duodenal feeding tubes beyond the second portion of the duodenum were placed for enteral nutrition and liquid food. At the age of 10 years, due to the appearance of a perianal abscess and gastric non-necrotizing granulomas with inflammatory cell infiltration (Figure 3A), the patient was diagnosed with Crohn's disease (CD). He received infliximab (eight times) and azathioprine treatments for CD in the following year. The level of serum infliximab did not achieve an adequate concentration (1 µg/ml) after three instances of intravenous infusions at a dose of 5 mg/kg. Then, the infliximab dose increased to 8 mg/kg, and the serum infliximab level increased to 3.5 µg/ml. The serum anti-infliximab antibody concentration was below 4 ng/ml. However, the infliximab infusion remained sub-therapeutic despite a dose escalation. The gastric lesions persisted, and the perianal abscess recurred after a partial improvement during the treatment. At the age of 12 years, upper gastrointestinal radiography showed gastric retention and severe pyloric stenosis (Figure 2C). The barium did not pass through the pylorus after 6 h (Figure 2D). A laparoscopic jejunostomy feeding tube placement was performed after 1 month of treatment with intravenous methylprednisolone. An EGD was placed through the surgical incision, and an intestinal biopsy showed normal intestinal villi without villous blunting or atrophy (Figure 3B). A gastrojejunal anastomosis bypass and jejunal-jejunal end-to-side anastomosis were performed 5 months later. The patient's vomiting improved; the patient was able to eat and this resulted in a significant weight gain after the surgery. At the age of 13 years, the patient had a height of 125 cm (Z-score, −4.21), weight of 29 kg (Z-score, −2.99), and BMI of 18.56 kg/m^2^ (Z-score, −0.06) (Supplementary Figure S1). Although the patient had a steady increase in height with age, his body weight fluctuated along with the recurrent symptoms and changes in nutrient intake. The patient's weight increased to 31.5 kg at the age of 13.5 years but dropped to 28.4 kg over the next 6 months due to the loss of appetite. At the age of 14 years, an EGD revealed persistent erosive gastritis with gastric ulcers, and a biopsy of the antrum showed significant eosinophil infiltration (40–65 eosinophils/HPF) (Figure 3C).

**Figure 4:**
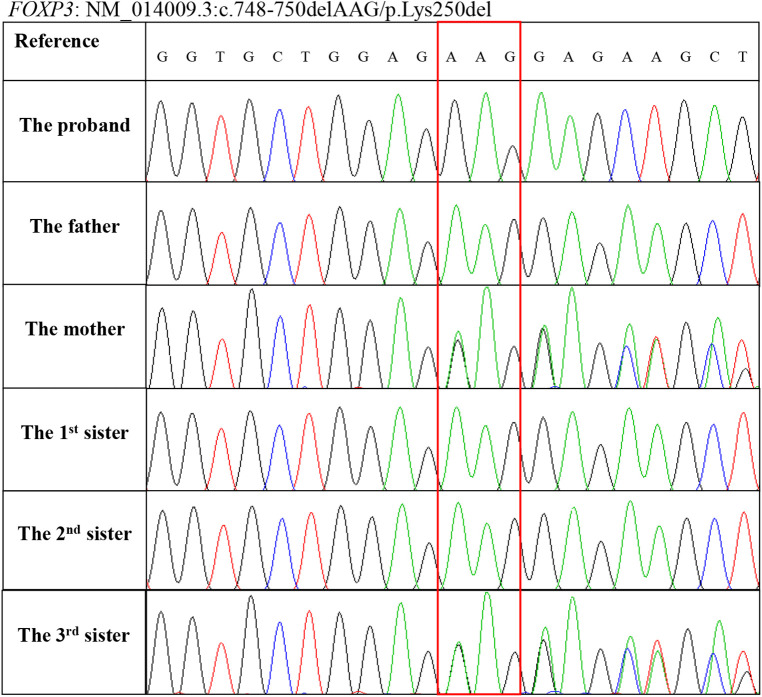
Genotyping of the *FOXP3* gene mutation c.748–750delAAG (p.Lys250del) in the family members using Sanger sequencing.

At this point, whole-exome sequencing (WES) was performed on a HiseqX10 (Illumina, USA) platform using genomic DNA extracted from the patient's peripheral blood. A 3 bp deletion in exon 7 of the *FOXP3* gene, c.748–750del (p.Lys250del), was identified and further confirmed by Sanger DNA sequencing. Genotyping of the unaffected family members by Sanger sequencing showed that his mother and third sister carry this mutation, and his father and two other sisters are normal (Figure 4). The c.748–750del (p.Lys250del) mutation is located in the leucine zipper domain of the FOXP3 protein, which is critical for dimerization and DNA binding, and a previous study demonstrated that the developments of the CD4^+^ CD25^+^ FOXP3^+^ T cell are significantly impaired in patients with this mutation (12). To date, intractable diarrhea and T1DM have not been observed.

The patient underwent matched sibling (his first sister) peripheral blood hematopoietic stem cell transplantation (HSCT) using reduced-intensity conditioning with busulfan, fludarabine, cyclophosphamide, and anti-thymocyte globulin 8 months after his genetic diagnosis. The CD34 cell dose was 0.37 × 10^6^/kg, the number of neutrophils was 0.75 × 10^9^/L, and the number of platelets was 300 × 10^9^/L. He received sustained cyclosporine (35 mg per day) and methylprednisolone (2 mg/kg per day) to prevent graft-versus-host disease (GvHD) 7 days before the HSCT. At day +17 after the HSCT, the short tandem repeat (STR) test showed that the patient was engrafted with 100% of donor cells. The patient started to experience a skin rash and diarrhea on day 26 after the HSCT. He was diagnosed with acute GvHD (grade 2) and treated with methylprednisolone and ruxolitinib. The skin rash disappeared, and the diarrhea recovered after 2 weeks of therapy. In addition, symptomatic treatments and antibiotics were applied to treat several episodes of oral, lung, and intestinal infections after the HSCT. The patient's weight decreased from 31 kg to 28 kg in 2 months after the HSCT. At the time of this writing, the patient is 15 years old and remains stable after 3 months of HSCT. The patient is managed with sustained cyclosporine (100 mg per day) and methylprednisolone (0.5 mg/kg per day). The patient takes food orally with a normal diet, and no feeding tube is needed. However, the patient continues to present with severe failure to thrive (height, 137 cm, Z-score, −4.01; weight, 28 kg, Z-score, −5.53; BMI, 14.88 kg/m^2^, Z-score, −3.16) (Supplementary Figure S1). Structuring and eosinophilic GI diseases should be re-evaluated shortly.

## Discussion

Classical IPEX syndrome caused by mutations in *FOXP3* is characterized by the triad of early-onset intractable diarrhea, type T1DM, and eczema (6). Emerging reported atypical cases in the last two decades, including late-onset symptoms, mild phenotypes, single system involvement, or rare clinical features, extend the clinical spectrum of IPEX syndrome (13). As an autoimmune disorder, symptoms other than the classical triad have been described, such as thyroiditis, thrombocytopenia, arthritis, neutropenia, nephropathy, and hepatitis (13). In this report, we described a case of atypical IPEX syndrome featuring eczema, food allergies, severe EG, and pyloric stenosis. The patient presented with early-onset eczema as an initial symptom in the first year of life. Food allergies were observed after the introduction of solid food. Vomiting, EG, pyloric stenosis, and failure to thrive developed at the age of 3 years. The patient's condition did not respond to long-term symptomatic therapies and nutritional support. Identical to several patients with reported atypical IPEX (14–17), the patient was suspected of having CD, but infliximab therapy was ineffective in improving the symptoms. The pyloric stenosis was further aggravated, and he had to receive a gastrojejunal anastomosis bypass. Finally, the patient was diagnosed with IPEX syndrome due to a 3 bp deletion in exon 7 of *FOXP3*, c.748–750del (p.Lys250del). This mutation is located in the leucine zipper domain of the FOXP3 protein and has been demonstrated to impair the development of the CD4^+^ CD25^+^ FOXP3^+^ T cell (12). The main reasons for the delayed genetic diagnosis of IPEX syndrome are due to the atypical clinical presentations and the parents’ reluctance for genetic testing. The patient was considered to have EG and CD-related pyloric stenosis. His brother's presentation of life-threatening diarrhea resulting in death at the age of 2 years elicited a possible family history of monogenic inborn immunity error. Genetic testing with WES was strongly recommended during his first hospitalization in our department at the age of 7 years. However, the patient's parents refused because he has three healthy sisters. Thus, physicians should be aware of the variable phenotypes of atypical IPEX syndrome. Early genetic testing is necessary to achieve an accurate diagnosis in cases of atypical IPEX, particularly in patients with a high degree of suspicion of family history. If a proband has healthy sisters, X-link defects must be considered in clinical practice.

Although enteropathy in IPEX syndrome can present different histopathologic phenotypes, such as GvHD-like changes, celiac disease-like pattern, and depletion of goblet cells (18), gastritis is rarely reported. Scaillon et al. reported a case of IPEX that developed with extensive exfoliative gastritis (19). Luo et al. reported a case of IPEX carrying the same mutation that presented with metaplastic atrophic gastritis and villus atrophy in the descending part of the duodenum (20). There have been several cases of eosinophilic esophagitis reported in IPEX syndrome (21) as well as at least two patients with pyloric stenosis described by Fang et al. (22), but it is rare to see chronic gastritis with eosinophilic infiltration (23). In this report, we described an atypical IPEX case presenting with severe gastritis and pyloric stenosis. The common histopathological finding of these IPEX cases with gastritis is remarkable eosinophil infiltration. Neither genotype-phenotype correlations nor phenotype associations have been observed in IPEX patients with gastritis. Four different mutations in *FOXP3*, c.1040G>A (R347H) (19), c.748–750del (p.Lys250del) (20), c.1280dupC (p.P427fs) (22), and c.766A>G (p.M256V) (22), have been identified in IPEX patients with gastritis. They can present simultaneously with either T1DM or other autoimmune manifestations. In addition, patients carrying the c.748–750del (p.Lys250del) mutation present with different clinical manifestations, such as nephrotic syndrome and autoimmune hepatitis (12, 20, 24). Therefore, further studies are needed to explore the genotype-phenotype correlations of IPEX syndrome. Nevertheless, a lack of enteropathy in atypical IPEX syndrome has been well reported in the literature (13). A recent review conducted by Consonni et al. showed that no enteropathy was described in 15 of 59 cases in a series of cases of atypical IPEX syndrome, reflecting approximately 25% (13).

The current therapeutic approaches for IPEX syndrome are immunosuppressive treatment and HSCT (6). Immunosuppression strategies can be effective, particularly in atypical IPEX cases with mild disease. HSCT is a curative therapy for IPEX syndrome, and the outcome depends on it being performed in the early stages of the disease (25). Thus, a prompt diagnosis is critical for managing patients with IPEX syndrome. Gastritis is an unusual gastrointestinal finding in IPEX syndrome, often leading to a delayed diagnosis of IPEX syndrome. Indeed, most of the reported IPEX cases with gastritis were diagnosed in adolescents by a confirmation of the *FOXP3* mutation by genetic testing (22). The delayed diagnosis of IPEX syndrome can lead to inappropriate therapy and an impaired quality of life in the patient. Pediatricians should raise awareness of IPEX syndrome and improve the diagnosis and intervention strategy.

In summary, this case report further highlights the unusual gastrointestinal findings in IPEX syndrome as well as the need for increased awareness as an early diagnosis of IPEX syndrome is vital for improving the patient's outcome.

## Data Availability

The datasets presented in this study can be found in online repositories. The names of the repository/repositories and accession number(s) can be found below: https://www.ncbi.nlm.nih.gov/, SRA/PRJNA888449.

## References

[1] Powell BR Buist NR Stenzel P. An X-linked syndrome of diarrhea, polyendocrinopathy, and fatal infection in infancy. J Pediatr. (1982) 100:731–7. 10.1016/s0022-3476(82)80573-8 7040622

[2] Chatila TA Blaeser F Ho N Lederman HM Voulgaropoulos C Helms C JM2, Encoding a fork head-related protein, is mutated in X-linked autoimmunity-allergic disregulation syndrome. J Clin Invest. (2000) 106:R75–R81. 10.1172/JCI11679 11120765 PMC387260

[3] Bennett CL Christie J Ramsdell F Brunkow ME Ferguson PJ Whitesell L The immune dysregulation, polyendocrinopathy, enteropathy, X-linked syndrome (IPEX) is caused by mutations of FOXP3. Nat Genet. (2001) 27:20–1. 10.1038/83713 11137993

[4] van der Vliet HJ Nieuwenhuis EE. IPEX As a result of mutations in FOXP3. Clin Dev Immunol. (2007) 2007:89017. 10.1155/2007/89017 18317533 PMC2248278

[5] Fontenot JD Gavin MA Rudensky AY. Foxp3 programs the development and function of CD4+CD25+regulatory T cells. Nat Immunol. (2003) 4:330–6. 10.1038/ni904 12612578

[6] Bacchetta R Barzaghi F Roncarolo MG. From IPEX syndrome to FOXP3 mutation: a lesson on immune dysregulation. Ann N Y Acad Sci. (2018) 1417:5–22. 10.1111/nyas.13011 26918796

[7] Chen PH Anderson L Zhang K Weiss GA. Eosinophilic gastritis/gastroenteritis. Curr Gastroenterol Rep. (2021) 23:13. 10.1007/s11894-021-00809-2 34331146

[8] Lwin T Melton SD Genta RM. Eosinophilic gastritis: histopathological characterization and quantification of the normal gastric eosinophil content. Mod Pathol. (2011) 24:556–63. 10.1038/modpathol.2010.221 21169993

[9] Talley NJ Shorter RG Phillips SF Zinsmeister AR. Eosinophilic gastroenteritis: a clinicopathological study of patients with disease of the mucosa, muscle layer, and subserosal tissues. Gut. (1990) 31:54–8. 10.1136/gut.31.1.54 2318432 PMC1378340

[10] Katiyar R Patne SC Dixit VK Sharma SP. Primary eosinophilic gastritis in a child with gastric outlet obstruction. J Gastrointest Surg. (2016) 20:1270–1. 10.1007/s11605-016-3074-6 26768007

[11] Leal R Fayad L Vieira D Figueiredo T Lopes A Carvalho R Unusual presentations of eosinophilic gastroenteritis: two case reports. Turk J Gastroenterol. (2014) 25:323–9. 10.5152/tjg.2014.6735 25141324

[12] Hashimura Y Nozu K Kanegane H Miyawaki T Hayakawa A Yoshikawa N Minimal change nephrotic syndrome associated with immune dysregulation, polyendocrinopathy, enteropathy, X-linked syndrome. Pediatr Nephrol. (2009) 24:1181–6. 10.1007/s00467-009-1119-8 19189134

[13] Consonni F Ciullini Mannurita S Gambineri E. Atypical presentations of IPEX: expect the unexpected. Front Pediatr. (2021) 9:643094. 10.3389/fped.2021.643094 33614561 PMC7892580

[14] Ge T Wang Y Che Y Xiao Y Zhang T. Atypical late-onset immune dysregulation, polyendocrinopathy, enteropathy, X-linked syndrome with intractable diarrhea: a case report. Front Pediatr. (2017) 5:267. 10.3389/fped.2017.00267 29312905 PMC5732958

[15] Duztas DT Al-Shadfan L Ozturk H Yazan H Cakir E Ekinci NUO New findings of immunodysregulation, polyendocrinopathy, and enteropathy X-linked syndrome (IPEX); granulomas in lung and duodenum. Pediatr Dev Pathol. (2021) 24:252–7. 10.1177/1093526621998868 33683986

[16] Yong PL Russo P Sullivan KE. Use of sirolimus in IPEX and IPEX-like children. J Clin Immunol. (2008) 28:581–7. 10.1007/s10875-008-9196-1 18481161

[17] Bindl L Torgerson T Perroni L Youssef N Ochs HD Goulet O Successful use of the new immune-suppressor sirolimus in IPEX (immune dysregulation, polyendocrinopathy, enteropathy, X-linked syndrome). J Pediatr. (2005) 147:256–9. 10.1016/j.jpeds.2005.04.017 16126062

[18] Patey-Mariaud de Serre N Canioni D Ganousse S Rieux-Laucat F Goulet O Ruemmele F Digestive histopathological presentation of IPEX syndrome. Mod Pathol. (2009) 22:95–102. 10.1038/modpathol.2008.161 18820676

[19] Scaillon M Van Biervliet S Bontems P Dorchy H Hanssens L Ferster A Severe gastritis in an insulin-dependent child with an IPEX syndrome. J Pediatr Gastroenterol Nutr. (2009) 49:368–70. 10.1097/MPG.0b013e3181a159de 19633572

[20] Luo Y Chen J Fang Y Lou J Yu J. A case of metaplastic atrophic gastritis in immune dysregulation, polyendocrinopathy, enteropathy, X-linked (IPEX) syndrome. BMC Pediatr. (2018) 18:191. 10.1186/s12887-018-1169-9 29907148 PMC6002972

[21] Tourangeau LM Doherty TA. Cardiopulmonary arrest in a patient with delayed diagnosis of immune dysregulation, polyendocrinopathy, enteropathy, X-linked syndrome. Allergy Asthma Proc. (2011) 32:74–8. 10.2500/aap.2011.32.3378 21262102

[22] Fang Y Luo Y Lou J Chen J. Atypical late-onset severe gastritis in immune dysregulation, polyendocrinopathy, enteropathy, and X-linked (IPEX) syndrome: 2 case reports. Medicine (Baltimore). (2021) 100:e24318. 10.1097/MD.0000000000024318 33546062 PMC7837857

[23] Huang Y Fang S Zeng T Chen J Yang L Sun G Clinical and immunological characteristics of five patients with immune dysregulation, polyendocrinopathy, enteropathy, X-linked syndrome in China-expanding the atypical phenotypes. Front Immunol. (2022) 13:972746. 10.3389/fimmu.2022.972746 36091011 PMC9448973

[24] Lopez SI Ciocca M Oleastro M Cuarterolo ML Rocca A de Davila MT Autoimmune hepatitis type 2 in a child with IPEX syndrome. J Pediatr Gastroenterol Nutr. (2011) 53:690–3. 10.1097/MPG.0b013e3182250651 21629128

[25] Barzaghi F Passerini L Bacchetta R. Immune dysregulation, polyendocrinopathy, enteropathy, x-linked syndrome: a paradigm of immunodeficiency with autoimmunity. Front Immunol. (2012) 3:211. 10.3389/fimmu.2012.00211 23060872 PMC3459184

