# Supplementary figures and images for: Case Report: Eosinophilic gastritis with pyloric stenosis in immune dysregulation, polyendocrinopathy, enteropathy, X-linked syndrome
Source: Front Pediatr. 2022 Nov 21;10:1039341. doi: 10.3389/fped.2022.1039341 (PMC9720107; doi:10.3389/fped.2022.1039341)

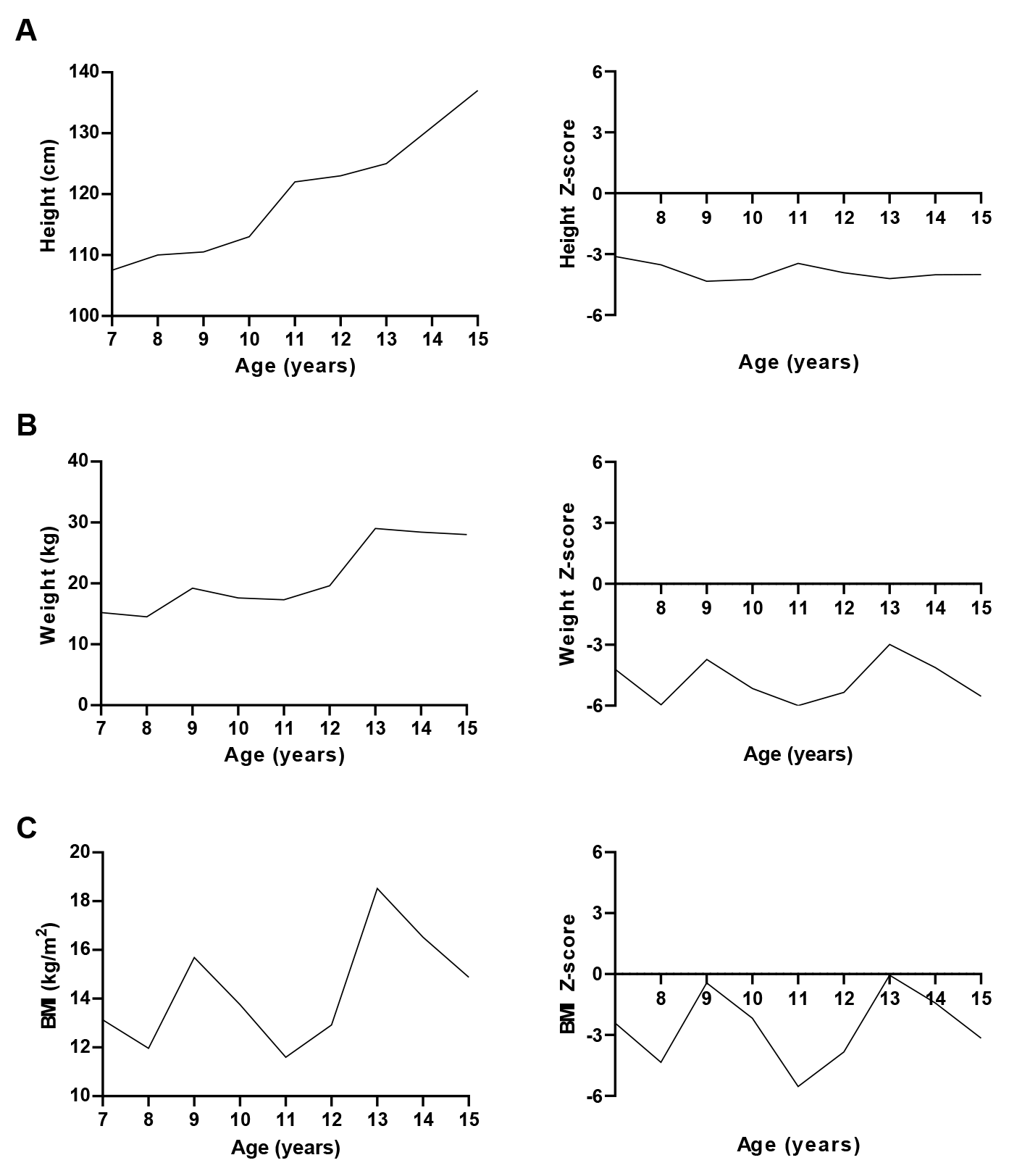

Supplement: Supplementary file 1 [file Image1.tif]
